# RAB42 overexpression correlates with poor prognosis, immune cell infiltration and chemoresistance

**DOI:** 10.3389/fphar.2024.1445170

**Published:** 2024-07-19

**Authors:** Yang Wang, Youbang Xie, Luomeng Qian, Ran Ding, Rongqing Pang, Ping Chen, Qing Zhang, Sihe Zhang

**Affiliations:** ^1^ Department of Cell Biology, School of Medicine, Nankai University, Tianjin, China; ^2^ Department of Hematology and Rheumatology, Qinghai Provincial People’s Hospital, Xining, Qinghai, China; ^3^ School of Biomedical Sciences and Engineering, South China University of Technology, Guangzhou International Campus, Guangzhou, China; ^4^ Basic Medical Laboratory, 920th Hospital of Joint Logistics Support Force, Kunming, Yunnan, China; ^5^ National Clinical Research Center for Cancer, Tianjin’s Clinical Research Center for Cancer, Tianjin Medical University Cancer Institute and Hospital, Tianjin, China

**Keywords:** RAB42, biomarker, tumor immune microenvironment, pan-cancer, hepatocellular carcinoma

## Abstract

**Background:**

RAB42 (Ras-related protein 42) is a new small GTPase that controls the vesicular trafficking from endosomes to trans-Golgi network in mammalian cells. However, the role of RAB42 in multiple cancers, especially in liver hepatocellular carcinoma (LIHC), has not been well investigated.

**Methods:**

A variety of cancer-related databases and online tools, including TCGA, GTEx, TARGET, QUANTISEQ, EPIC, RNAactDrug, CTR-DB, TIMER algorithms and Sangerbox, were applied to explore the correlation of RAB42 expression with prognosis, immune microenvironment, immune regulatory network, RNA modification, pathway activation and drug sensitivity in pan-cancer. The prognostic, immunomodulatory and tumor-promoting effects of RAB42 were verified in various malignancies and determined by a series of *in vitro* cellular experiments.

**Results:**

RAB42 is significantly overexpressed in most cancers with advanced pathological stages. Its overexpression is correlated with poor survival in pan-cancer. RAB42 overexpression has a high diagnostic accuracy of various cancers (AUC > 0.80). RAB42 overexpression not only correlates with distinct stromal immune infiltration and level of immune checkpoint molecules, but also associates with weak immune cell infiltration, immunomodulatory genes expression, and immunotherapeutic response to immune checkpoint inhibitors (ICIs). Additionally, RAB42 overexpression correlates with enhanced expression of m6A RNA methylation-related genes (MRGs) and its interactors. Moreover, overexpression of RAB42 serves as a drug-resistant marker to certain chemotherapies and acts as a potential biomarker for LIHC. Notably, RAB42 overexpression or activation promotes the cellular proliferation, migration and invasion of LIHC.

**Conclusion:**

Overexpressed RAB42 serves as a potential prognostic biomarker and therapeutic target in pan-cancer, especially in LIHC.

## Introduction

RAB (Ras-related proteins) constitute the largest family of small GTPases in Ras superfamily. More than 60 RAB GTPases have been identified in human tissues (Borchers et al., 2021; Homma et al., 2021). RAB GTPases drive the intercellular vesicle trafficking. They control the formation of membrane buds, vesicular transport, and membrane fusion via transient interacting with their downstream effectors (Jin et al., 2021; Wilmes and Kümmel, 2023). As the key GTPases, activated RAB proteins boost the endocytosis, degradation, recycling and exocytosis of vesicles through complex mechanisms. Dysregulated expression of RAB proteins is tightly associated with malignances (Tzeng and Wang, 2016; Jin et al., 2021). Furthermore, overexpression of certain RAB GTPase can augment the proliferation, invasion and migration of some cancer cell types (Yang C. C. et al., 2021). For example, RAB9A overexpression promotes the proliferation and invasion of liver hepatocellular carcinoma (LIHC) cells (Sun et al., 2020). RAB14 overexpression promotes the migration, invasion and metastasis of LIHC cells by regulating the transport of Mucin13 and CD44 (Chen et al., 2019). Our previous works showed that activation of RAB5 and RAB22 promotes the CD147-mediated invasion of LIHC cells (Qi et al., 2019a; Qi et al., 2019b). These findings support the indispensable role of RAB GTPases in promoting the malignant phenotypes of cancers.

RAB42 (Ras-related protein 42) is a new member of the RAB GTPase family. However, its function in vesicle trafficking is unclear. Recently, two works have implied that it may associate with activated VEGF signaling in glioma and inhibited E2F signaling in LIHC (Liu et al., 2021; Kong et al., 2023). Another two works have suggested that RAB42 may act as a pro-oncogene that drives tumor progression and may be a new target associated with immunotherapy in certain tumor types (Peng et al., 2022; Zheng et al., 2022). Although these studies preliminarily uncovered that genetic and epigenetic factors control RAB42 overexpression and implied its clinical prognostic potential, its detailed functions in pan-cancer are rarely investigated. Bioinformatics-based pan-cancer analysis can provide a panoramic overview, comprehensive evidence, and prospective direction for targets of interest. By integrating the multiomics datasets from public databases, pan-cancer analysis enables a more comprehensively understanding of the molecular mechanism, genotype-phenotype connection, and predictive value of a target protein, thereby maximizing the options for clinical diagnosis and treatment (Liu H. et al., 2022; Connor and Gallinger, 2022; Li Y. et al., 2023).

In this study, we conducted a pan-cancer analysis of RAB42 expression, and investigated its prognostic, immunotherapeutic and chemoresistance-predictive values. We found that RAB42 overexpression correlates with poor clinical prognosis and can be used to diagnose most types of cancers. RAB42 overexpression also correlates with enhanced RNA modifications, poor tumor immune cell infiltration, and declined expression of immune checkpoint genes in cancers that are non-response to immune checkpoint inhibitors (ICIs). Notably, RAB42 functions as a marker to predict drug resistance in certain chemotherapies and serves as a biomarker for LIHC. Modulating RAB42 expression or activation via the knockdown or mutant-overexpression approaches can regulate cellular proliferation, invasion and migration of LIHC. Taken together, these findings suggest that RAB42 serves as both a potential prognostic marker and a therapeutic target for pan-cancer, especially for LIHC.

## Materials and methods

### Pan-cancer data acquisition

The collated data of 11,641 TCGA pan-cancer samples and 10,406 samples integrated with GTEx samples (https://commonfund.nih.gov/GTEx/) were downloaded from the UCSC Xena database. Standardized transformation [log2(x+0.001)] was performed, including mRNA sequencing data for RAB42 in 34 different types of tumor tissues and 31 normal tissues with relevant clinical data (survival status, clinical and pathological stage. https://doi.org/10.1002/imt2.36). During this process, cancers with fewer than 3 samples were discarded. In addition, R packages “limma”, “ggplot2” and “ggpubr” were used to analyze the expression of RAB42 in cancer tissues. TIMER 2.0 platform was used to acquire and visualize the RAB42 expression in cancer tissues (http://timer.cistrome.org/). The abbreviations for different tumor types are shown (Supplementary Table S1).

### Prognostic assessment for pan-cancer

By deleting the samples with incomplete information on pan-cancer expression data and clinical information, high-quality prognostic data were collated. A univariate Cox regression model was constructed by using the R package “survival”. The prognostic value of RAB42 for cancer patients was assessed by 4 clinical outcomes: overall survival (OS), disease-specific survival (DSS), progression-free survival (PFS) and disease-free survival (DFS). Prognostic indicators, including hazard ratios (HRs), 95% confidence intervals (95% CIs) and *p* values, were calculated under standard conditions (Zhang et al., 2022; Su et al., 2024). A result was considered significant when *p* < 0.05.

### Correlation analysis of RAB42 expression with clinical stage

Correlation between clinical stage and RAB42 expression was analyzed by the R packages “limma” and “ggpubr”. Violin plots were drawn by using GEPIA online tools (http://gepia.cancer-pku.cn/). R packages “pROC” and “ggplot2” were used to determine the receiver operating characteristic (ROC)-area under the curve (AUC) values of RAB42. In principle, AUC values greater than 0.8 are considered to be high reliability (Zhang et al., 2022).

### Correlation analysis of RAB42 expression with immune traits

The gene expression profiles of RAB42, 60 immune checkpoint genes, 41 chemokines, 18 chemokine receptors and 21 major histocompatibility complex (MHC)-related immune signatures across different types of cancers were collected from a standardized gene expression dataset (Thorsson et al., 2018). Correlation analyses were performed by using the R package “limma”, and correlation coefficients were determined via the Pearson statistical approach. The stromal, immune, and ESTIMATE scores were calculated for 10,180 tumor samples. All the data were collected from 44 tumor types and visualized by the R package “ESTIMATE” (Yoshihara et al., 2013). Spearman correlation between RAB42 expression and immune cell infiltration scores across various cancer types was measured by the R package “psych”. Then, immune infiltration scores were calculated. Immune cell infiltration data for 33 cancers were downloaded from the TIMER 2.0 database (http://timer.cistrome.org) (Li et al., 2020). Infiltration scores of B cells, T cells, neutrophils, macrophages, DCs, and other immune cells across various cancer types were calculated by using the “Timer,” “deconvo_epic,” and “deconvo_quantizeq” algorithms (Racle et al., 2017; Finotello et al., 2019; Zeng et al., 2021). The results were correspondingly visualized by the R packages “IOBR,” “reshape2” and “RColorBreyer”.

### Correlation analysis of RAB42 expression with genomic heterogeneity and stemness

Homologous recombination deficiency (HRD) scores and tumor purity data for all cancer types were obtained from a previous study (Thorsson et al., 2018). The collated gene expression data were integrated with HRD and loss of heterozygosity (LOH) data from the pan-cancer samples, and Spearman correlation analyses were performed. Processed samples for diverse cancers were downloaded from the TCGA and GDC online databases (https://portal.gdc.cancer.gov/), and subjected to correlation analyses (Beroukhim et al., 2010). Tumor mutational burden (TMB) scores were calculated by using the “maftools” R package. Stemness scores were calculated by the OCLR algorithm (Malta et al., 2018). Stemness scores and gene expression data of all samples were integrated together for Pearson correlation analyses and visualization.

### Expression correlation analysis of RAB42 with m6A RNA methylation-related genes (MRGs)

Standardized pan-cancer data (PANCAN, *n* = 19,131, g = 60,499) were downloaded from the TCGA, TARGET and GTEx databases. The expression data of RAB42 and 21 m6A RNA MRGs in various samples were extracted. The expression correlation between RAB42 and m6A RNA MRGs was calculated and visualized via the Sangerbox.

### Drug sensitivity analysis

To explore the predictive value of RAB42 expression on drug-resistance, RNAactDrug Database (http://bio-bigdata.hrbmu.edu.cn/RNAactDrug/) and Cancer Treatment Response gene signature DataBase (CTR-DB) (http://ctrdb.cloudna.cn/home) were employed to analyze the drug sensitivity in pan-cancer (Dong et al., 2020; Liu Z. et al., 2022). The predictive accuracy of RAB42 expression on chemoresistance was determined based on the AUC values (Su et al., 2024).

### Cell culture, siRNA transfection and plasmid overexpression

The human LIHC cells MHCC-97H and HepG2, obtained from the Type Culture Collection of the Chinese Academy of Sciences (China), was routinely cultured (37°C, DMEM with fetal bovine serum, 10% v/v) in normoxic (5% CO2) incubators (Thermo). Cultured LIHC cells were transiently transfected with RAB42-targeting siRNA oligos or RAB42-coding plasmid DNA (pDNA) for 12 h using Lipofectamine^®^ 2000 (Invitrogen), and continued culture for 48 h for use in assays (Qi et al., 2019a; Qi et al., 2019b). RAB42-targeting siRNA sequences: siRNA#1: 5′-CCA​GGU​CCU​UUU​ACC​GGA​A-3’; siRNA#2: 5′-GGA​AGU​CCU​UUG​AAC​ACA​U-3′. Scrambled siRNA was used as a negative control (NC). EGFP-fused RAB42 plasmids, wild type RAB42(wt), activated mutant RAB42(Q76L), dominant-negative mutant RAB42(H129I), were supplied by Prof. Guangyu Wu (Augusta University) and Chunman Li (Shantou University) (Wang and Wu, 2012; Li et al., 2017; Wei et al., 2019).

### Western blotting

Cultured LIHC cells were washed with cold PBS and then lysed in standard RIPA lysis buffer (10 mM Tris-HCl, pH 7.4, 150 mM NaCl, 1 mM EDTA, 0.1% SDS, and 1% Triton X-100). After sonication for three times (2 s each cycle), cell debris was removed via centrifugation at 10,000 rpm for 10 min, and protein concentration of the supernatant was measured by using a BCA Kit (Thermo Scientific). The protein samples were resolved via SDS‒PAGE, blotted onto PVDF membranes and subsequently incubated with primary Abs (anti-RAB42 Ab, 1:100; sc-130482. Santa Cruz; anti-GFP Ab, 1:1000, M20004, Abmart; anti-Beta actin Ab, 1:20000, 66009-1, Proteintech) overnight at 4°C. After incubating with corresponding secondary Abs, the expression of target proteins was visualized by ECL reagents.

### CCK-8 assay

Proliferation of LIHC cells was examined by using CCK-8 kits (BMU106, SuperKine) according to the manufacturer’s instructions. siRNA- or pDNA-transfected LIHC cells were seeded in 96-well plates (2 × 10^3^ cells/well) and cultured for 48 h at 37°C. After that, CCK-8 solution (10 μL/well) was added and incubated for 30 min, the absorbance (450 nm) in each well was measured by a microplate reader (FLUOstar Omega; BMG Labtech).

### Scratch-migration assay

LIHC cells with/without siRNA- or pDNA-transfection were grown to a monolayer in 12-well plates (0.6% gelatin coated) and starved overnight with DMEM containing 0.1% FBS. After the cell monolayer was scraped with a sterile micropipette tip, complete medium (10% FBS) was added (*t* = 0). The cells were photographed at different time points under a phase-contrast microscope, and the wounded area was measured and calculated using ImageJ software.

### Matrigel-coated transwell invasion assay

LIHC cells with or without siRNA- or pDNA-transfection were harvested and seeded (1 × 10^5^) in matrigel-coated transwell inserts (serum-free medium. 8 μm pore size, BK Falcon). The lower chamber was filled with 10% FBS medium. After 24 h of incubation, cells on the filter of upper chamber were removed with a cotton swab, and cells on the underside were stained with a crystal violet solution and counted under a microscope (Qi et al., 2019a; Qi et al., 2019b). The number of invading cells was calculated by ImageJ.

### Patient samples and immunohistochemistry (IHC)

Ten fresh surgical specimens were collected from LIHC patients, and the informed consent was obtained from the Review Board of Nankai University (NKUIRB2021043). IHC staining was performed as previously described (Qi et al., 2019a; Qi et al., 2019b; Li et al., 2022; Ye et al., 2024). Briefly, after deparaffinization, rehydration, and antigen-retrieval treatment with 10 mM sodium citrate buffer (pH 6.0) at 120°C for five minutes, the slides were treated with hydrogen peroxide to quench endogenous peroxidase, blocked with goat serum, and incubated with anti-RAB42 Ab (1:20; sc-130482. Santa Cruz) overnight at 4°C. Goat anti-mouse IgG-HRP was used as a secondary Ab and normal mouse serum was used as a NC. IHC staining was performed with an EnvisionTM two step system (Dako, USA). The antibody-based IHC staining signal was measured and calculated by using ImageJ software.

### Statistical analysis

Comparison of gene expression difference was performed by using the Wilcoxon rank sum and Kruskal–Wallis tests. Spearman or Pearson analysis was used to evaluate the correlation between two groups. K-M and Cox regression analyses were used to analyze the survival characteristics. The Chi-square test and Fisher’s exact test were applied to analyze the clinical characteristics. Statistical analysis and visualization were performed by using the R and GraphPad Prism 9 software. *p* < 0.05 (*), *p* < 0.01 (**) and *p* < 0.001 (***) were considered significant.

## Results

### RAB42 is overexpressed in pan-cancer with advanced stages

**FIGURE 1:**
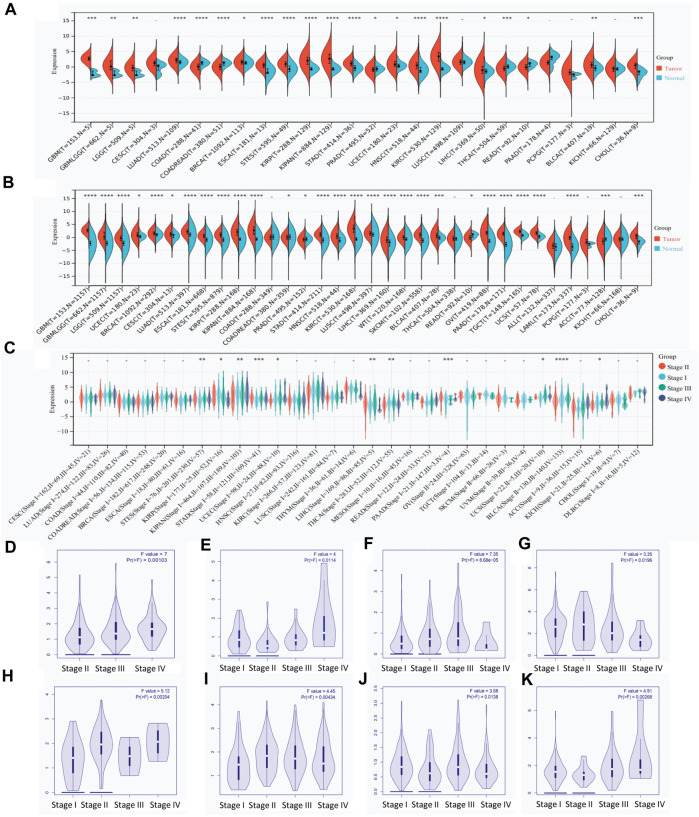
RAB42 overexpression in pan-cancer with advanced clinical stages. **(A)** Expression of RAB42 in pan-cancer and normal tissues derived from the TCGA dataset. **(B)** Expression of RAB42 in pan-cancer and normal tissues derived from the combined TCGA and GTEx datasets. **(C)** Expression of RAB42 at distinct clinical stages of pan-cancer. **(D–K)** RAB42 expression at different clinical stages of BLCA, KICH, LIHC, LIRP, PAAD, STAD, THCA, and UCEC. *p*-value: early stages (I + II) compared to advanced stages (III + IV). ns, *p* ≥ 0.05; *, *p* < 0.05; **, *p* < 0.01; ***, *p* < 0.001; ****, *p* < 0.0001.

By combining the data from TCGA and GTEx databases, the expression level of RAB42 across various types of cancers was visualized. RAB42 is overexpressed in 18 malignancies (Figure 1A), predominantly in GBM, LUAD, ESCAT, STES, KIRP, KIPAN, STAD, HNSC, KIRC, THCA, and CHOL. When the number of samples increased, RAB42 is overexpressed in almost all malignancies including LIHC (Figure 1B). The significant difference of RAB42 expression is observed in GBM (*p* = 1.1e-84), KIRC (*p* = 2.5e-71), LAML (*p* = 2.7e-64), and OV (*p* = 5.1e-45). Further pan-cancer analyses showed that the expression level of RAB42 significantly differs among distinct pathological stages. In STES, KIRP, KIPAN, STAD, UCEC, LIHC, THCA, PAAD, UCS, BLCA and KICH cancers, overexpression of RAB42 frequently occurs in stages III and IV (Figure 1C). Next, we analyzed the RAB42 expression level in GEPIA database. RAB42 overexpression is observed in advanced stages (III + IV) of 8 malignancies (Figures 1D–K). These results suggested that RAB42 is overexpressed across cancers with advanced pathological stages.

### RAB42 overexpression correlates with poor survival in pan-cancer

**FIGURE 2:**
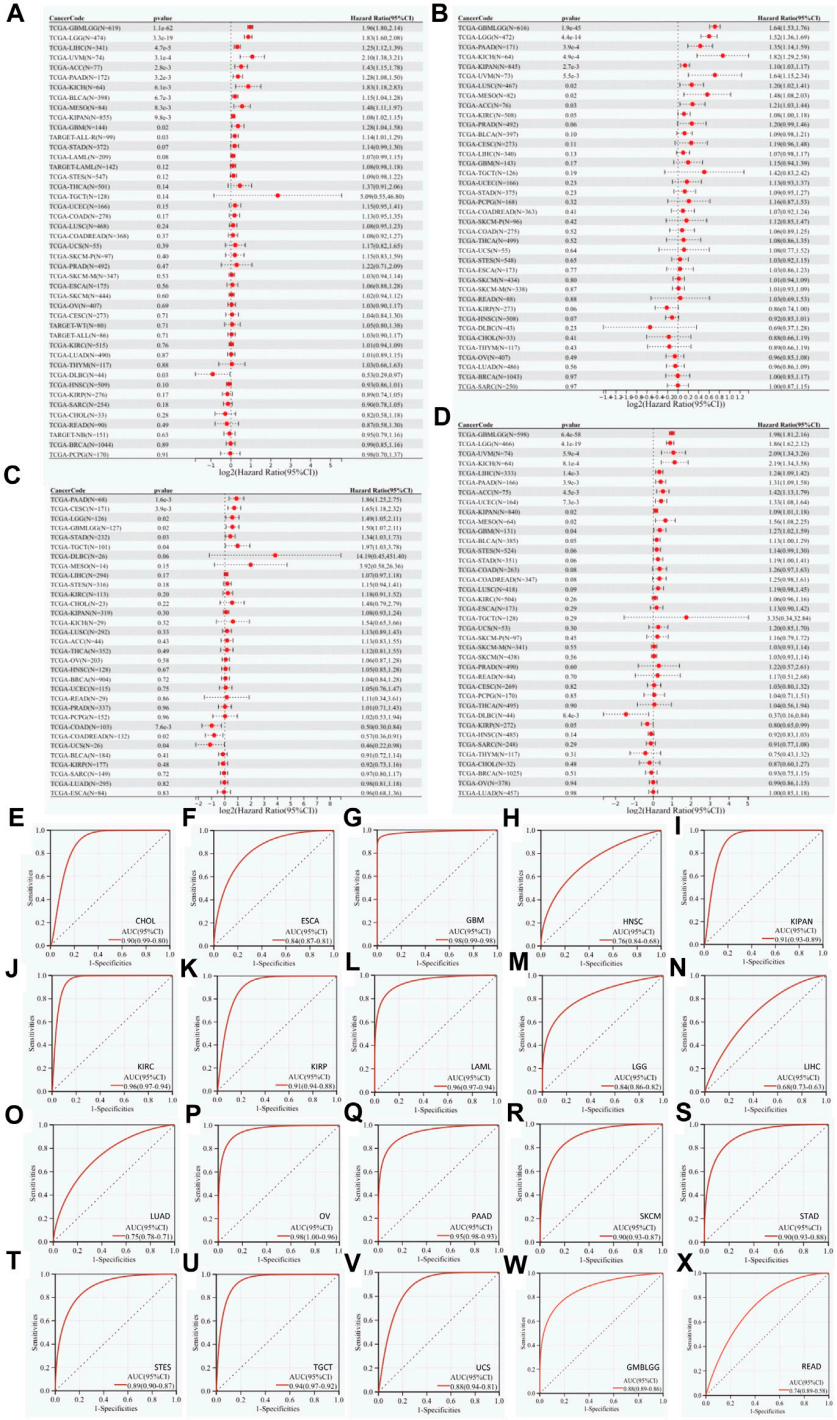
Prognostic and diagnostic value of RAB42 overexpression in pan-cancer. Correlation analyses between RAB42 expression and **(A)** OS, **(B)** PFS, **(C)** DFS, and **(D)** DSS in pan-cancer. **(E–X)** ROC curves-based diagnostic accuracy analysis of RAB42 expression in pan-cancer.

Univariate Cox regression analyses showed that RAB42 overexpression is a risk factor for short OS of 12 malignancies: GBMLGG, LGG, LIHC, UVM, ACC, PAAD, KICH, BLCA, MESO, KIPAN, GBM, and ALL-R (Figure 2A). Further analyses showed that RAB42 overexpression is markedly associated with short PFS in 9 malignancies: GBMLGG, LGG, PAAD, KICH, KIPAN, UVM, LUSC, MESO, and ACC (Figure 2B). In addition, RAB42 overexpression is significantly associated with short DFS in PAAD, CESC, LGG, GBMLGG, STAD and TGCT (Figure 2C) and poor prognosis in 11 malignancies: GBMLGG, LGG, UVM, KICH, LIHC, PAAD, ACC, UCEC, KIPAN, MESO and GBM (Figure 2D). Prognostic value of RAB42 for OS, DSS, DFS and PFS were demonstrated by Kaplan-Meier (KM) survival curves, with similar results of univariate Cox regression analyses. In patients with GBMLGG, LGG, LIHC, UVM, ACC, PAAD, KICH, BLCA, MESO and GBM, RAB42 overexpression is significantly associated with short OS (Supplementary Figure S1). In patients with GBMLGG, LGG, PAAD, KICH, KIPAN, UVM, LUSC, MESO and ACC, RAB42 overexpression is significantly associated with short PFS (Supplementary Figure S2). KM survival analyses showed that RAB42 overexpression is significantly associated with short DFS in patients with PAAD, CESC, LGG, GMBLGG, STAD and TGCT (Supplementary Figure S3). Again, RAB42 overexpression is significantly associated with short DSS in patients with GBMLGG, LGG, UVM, KICH, LIHC, PAAD, ACC, UCEC, KIPAN, GBM, and MESO (Supplementary Figure S4). ROC curves for pan-cancer were subsequently plotted, and the plots showed that RAB42 overexpression exerts diagnostic potential (AUC > 0.8) for 16 malignancies: CHOL (AUC = 0.90), ESCA (AUC = 0.84), GBM (AUC = 0.98), LGG (AUC = 0.84), OV (AUC = 0.98), PAAD (AUC = 0.95), KIPAN (AUC = 0.91), KIRC (AUC = 0.96), KIRP (AUC = 0.91), LAML (AUC = 0.96), SKCM (AUC = 0.90), STAD (AUC = 0.90), STES (AUC = 0.89), TGCT (AUC = 0.94), UCS (AUC = 0.88), and GMBLGG (AUC = 0.88) (Figures 2E–X). These results suggested that RAB42 overexpression has highly clinical prognostic and diagnostic value across various types of cancers.

### RAB42 overexpression correlates with distinct stromal immune infiltration and checkpoint level

**FIGURE 3:**
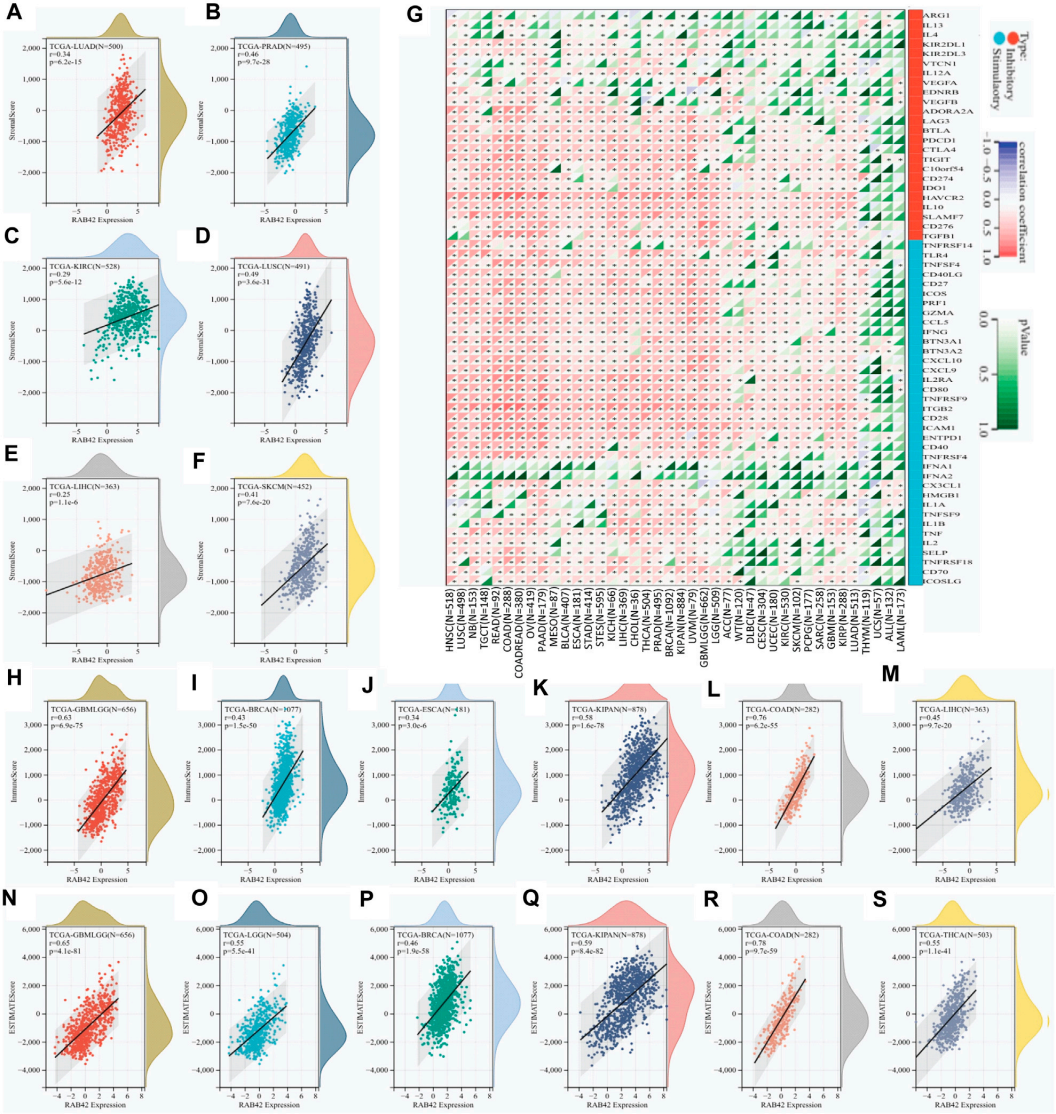
RAB42 overexpression correlates with immune cell infiltration and immune checkpoint expression. **(A–F)** Correlation analysis of RAB42 expression with stromal scores in selected cancers. **(G)** Expression correlation analysis between RAB42 and immune checkpoint-related genes in pan-cancer. **(H–M)** Correlation analysis of RAB42 expression with immune scores in selected cancers. **(N–S)** Correlation analysis of RAB42 expression with ESTIMATE scores in selected cancers.

To explore the role of RAB42 in tumor immune microenvironment (TIME), the correlation between RAB42 expression and three immune scores (stromal, immune, and estimated) were analyzed. The stromal scoring showed that RAB42 overexpression is positively correlated with stromal cell infiltration in LUAD, PRAD, KIRC, LUSC, LIHC, and SKCM (Figures 3A–F). The immune scoring showed that RAB42 overexpression is positively correlated with increased immune cell infiltration in GBMLGG, BRCA, ESCA, KIPAN, COAD, and LIHC (Figures 3H–M). ESTIMATE scoring showed that RAB42 overexpression is significantly correlated with the stromal and immune cell infiltration in GBMLGG, LGG, BRCA, KIPAN, COAD, and THCA (Figures 3N–S). Expression correlation analyses between RAB42 and immune checkpoint genes were subsequently analyzed. The results showed that RAB42 overexpression is significantly correlated with that of partial immunosuppressive genes (C10orfs54, CD274, IDOI, HAVCR2, IL10, SLAMF7, CD276, TGFB1) and immunostimulatory genes (TNFRSF14, TLR4, TNFSF4, CD40LG, CD27, ICOS, PRF1, GZMA, CCL5, IFNG, BTN3A1, BTN3A2, CXCL10, CXCL9, IL2RA, CD80, TNFRRSF9, ITGB2, CD28, ICAM1, ENTPD1, CD40, TNFRSF4) across various cancer types. Notably, overexpression of RAB42 is positively correlated with that of most immune checkpoint genes in HNSC, COADREAD, OV, PAAD, LIHC, and LUAD. However, its overexpression is negatively correlated with that of immune checkpoint genes in several THYM-included cancers (Figure 3G). Although these results differ slightly, the overall correlation analyses suggested that RAB42 overexpression is associated with TIME across various cancer types.

### RAB42 overexpression correlates with weak immune cell infiltration, immunomodulatory genes expression, and immunotherapeutic response to ICIs

**FIGURE 4:**
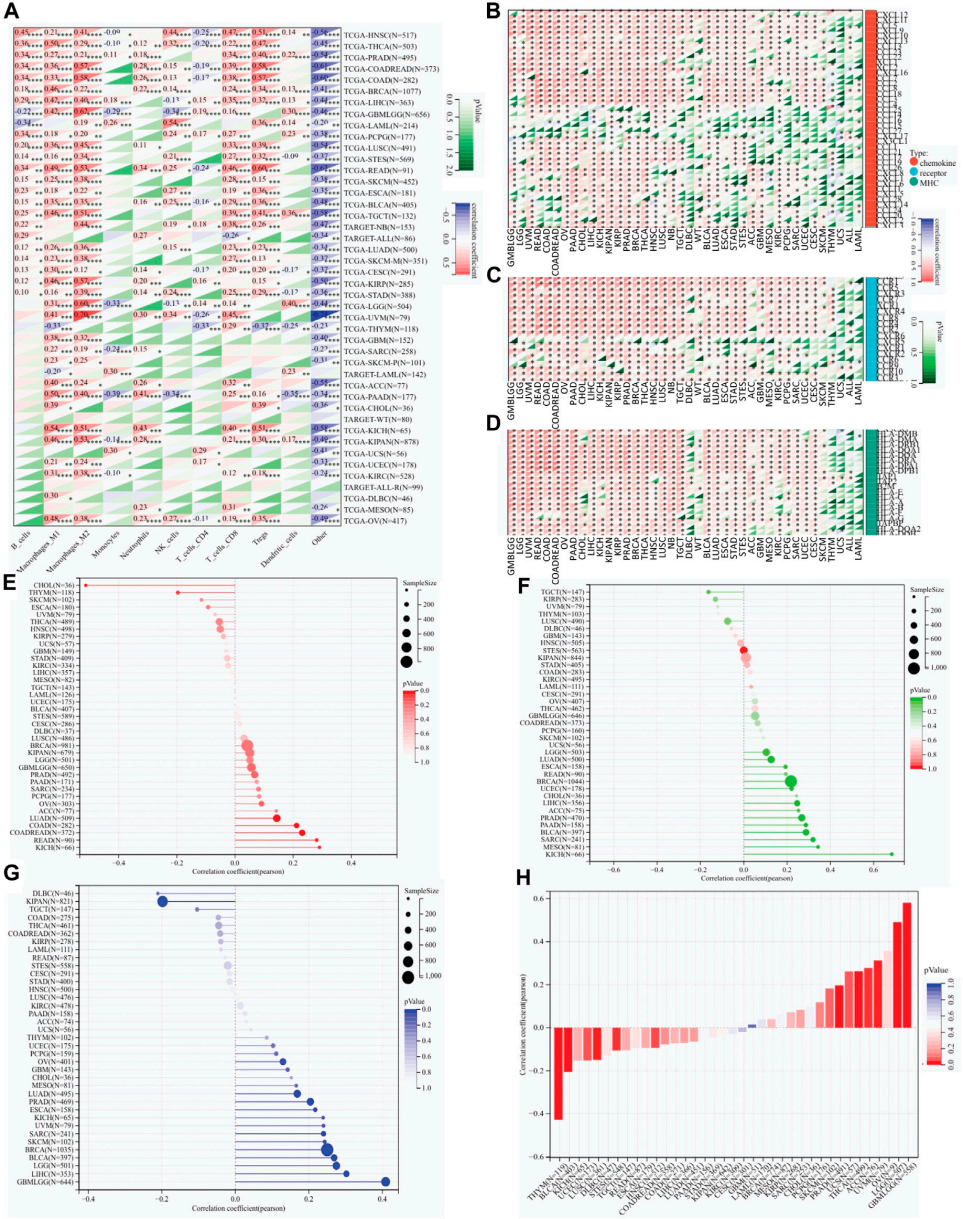
RAB42 overexpression correlates with the infiltration of immune cells. **(A)** Correlation analysis of RAB42 expression with immune cell infiltration in pan-cancer by predicting the treatment response to ICIs. A QUANTISEQ algorithm. *p < 0.05; **p < 0.01; ***p < 0.001; ****p < 0.0001. **(B–D)** Expression correlation analysis of RAB42 with immunomodulatory genes: chemokines **(B)**, chemokine receptors **(C)**, and MHCs **(D)**. **(E–H)** Correlation analysis of RAB42 expression with **(E)** TMB, **(F)** HRD, **(G)** LOH and **(H)** stemness scores.

The QUANTISEQ, EPIC and TIMER are three well-established algorithms frequently used to evaluate the immune scores for tumors. We next used these algorithms to analyze the correlation between RAB42 overexpression and infiltrated immune cells across various types of cancers. RAB42 overexpression is significantly correlated with weakly infiltrated macrophages, CD8^+^ and CD4^+^ T cells, neutrophils, Tregs and dendritic cells across various cancer types (Figure 4A; Supplementary Figures S5A, B). Further analyses showed that RAB42 overexpression is significantly correlated with increased infiltrated immune cells in HNSC, THCA, BRCA, LIHC, PRAD, COADREAD and COAD (Figure 4A). Notably, overexpression of RAB42 is most positively correlated with the infiltration of M2 macrophage and Treg in COADREAD, COAD, READ, and KICH cohorts (Figure 4A; Supplementary Figures S5A, B). The expression correlation of RAB42 with immunomodulatory-related genes, including MHC, chemokine and chemokine receptor, were further analyzed. RAB42 overexpression is positively correlated with that of the three gene families in most cancer types (Figures 4B–D). Notably, overexpression of RAB42 is positively correlated with that of these gene families in 12 malignancies. In COADREAD, COAD, OV, PAAD, LIHC, PRAD, THCA, and NB, RAB42 overexpression is positively correlated with that of almost all MHC and chemokine receptor genes. Such evident correlations are not found for DLBC, WT, ACC, THYM, UCS, ALL, or LAML (Figures 4B–D). In contrast, RAB42 overexpression is not significantly correlated with the expression of several chemokine genes (CCL25, CCL14, CCL16, CCL15 and CXCL17) in all malignancies. As TMB and HRD are key factors for immune checkpoint therapy (Georgiadis et al., 2019; Silva et al., 2022), the correlation between RAB42 expression and TMB or HRD contents were analyzed. Overall, RAB42 overexpression is positively correlated with the TMB and HRD across various cancer types. In KICH, READ, COADREAD and COAD cohorts, TMB scores are positively correlated with the RAB42 overexpression (Figure 4E), while HRD scores are positively correlated with the RAB42 overexpression in KICH, MESO, SARC, BLCA, PAAD, PRAD, ACC, LIHC, CHOL, UCEC and BRCA cohorts (Figure 4F). In addition, RAB42 overexpression is negatively correlated with TMB in CHOL and THYM cohorts (Figure 4E). LOH also affects the efficacy of ICIs treatment (Labriola et al., 2020). RAB42 overexpression is positively correlated with LOH contents in GBMLGG, LIHC, LGG, BLCA, BRCA, SKCM, SARC, UVM, KICH, ESCA and PRAD, whereas it is negatively correlated with LOH contents in DLBC and KIPAN (Figure 4G). Stemness scores were reported to correlate with cancer cell proliferation and drug resistance (Glinsky, 2008). Correlation analyses showed that RAB42 overexpression is significantly correlated with stemness scores in 11 malignancies. Notably, highly positive correlation of RAB42 overexpression and stemness scores is observed in 7 malignancies (GBMLGG, LGG, OV, UVM, ACC, THCA, and UCS). In contrast, highly negative correlation is observed in THYM and BLCA (Figure 4H). These results suggest that RAB42 overexpression significantly reshapes TIME in pan-cancer.

### RAB42 overexpression correlates with enhanced expression of m6A RNA MRGs and its interactors

**FIGURE 5:**
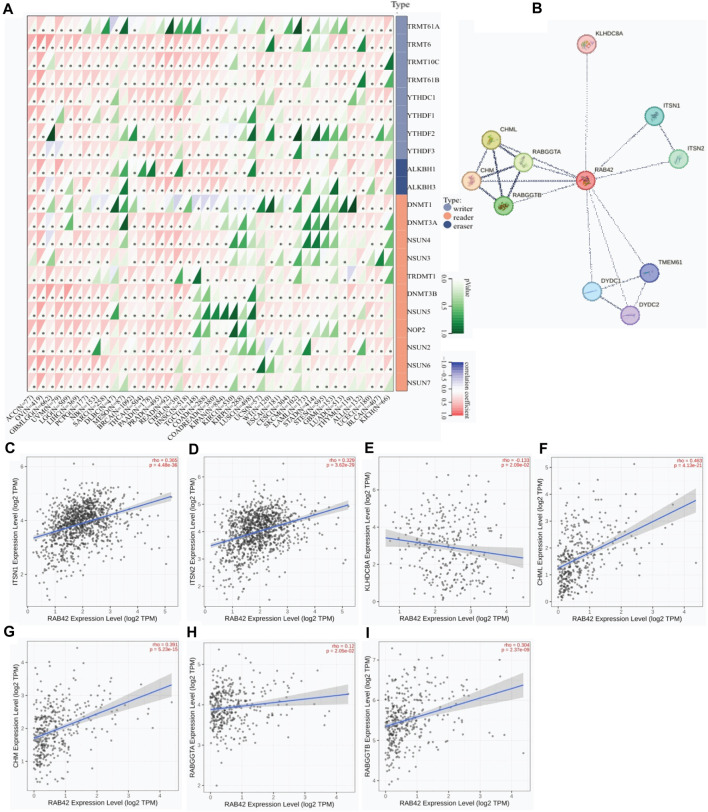
Protein-protein interaction network analysis of RAB42 in pan-cancer. **(A)** Expression correlation analysis of RAB42 with m6A RNA MRGs. *p < 0.05. **(B)** PPI network analysis of RAB42 in pan-cancer. **(C,D)** Expression correlation analysis of RAB42 with ITSN1 or ITSN2 in BRCA. **(E)** Expression correlation analysis of RAB42 with KLHDC8A in OV. **(F–I)** Expression correlation analyses of RAB42 with CHML, CHM, RABGGTA and RABGGTB in LIHC.

As dysregulated expression of m6A RNA MRGs is contributed to tumorigenesis, the expression correlation between RAB42 and m6A RNA MRGs was analyzed. Among the fifteen of forty malignancies, RAB42 overexpression is positively correlated with that of most m6A RNA MRGs, namely, writers, readers and erasers (Figure 5A). RAB42 overexpression is positively correlated with all m6A RNA MRGs in ACC, UCEC, PCPG, PRAD, OV and LIHC. Whereas, no significant expression correlation between RAB42 and most m6A RNA MRGs is observed in DLBC, SKCM, LAML, GBM and KICH (Figure 5A). Further analyses revealed that RAB42 overexpression is positively correlated with writer genes TRMT10C, TRMT61B and reader gene DNMT3B in most cancers. Although such correlation is differed in certain cancers, the whole expression correlation of RAB42 with m6A RNA MRGs is evident in pan-caner. Furthermore, PPI network analysis showed that RAB42 could directly interact with 11 proteins in cells (Figure 5B). Further correlation analyses indicated that RAB42 overexpression positively correlates with that of ITSN1, ITSN2, CHML, CHM, and RABGGTB in BRCA, OV and LIHC (Figures 5C–I).

### RAB42 overexpression predicts the response to certain chemotherapeutic drugs

**FIGURE 6:**
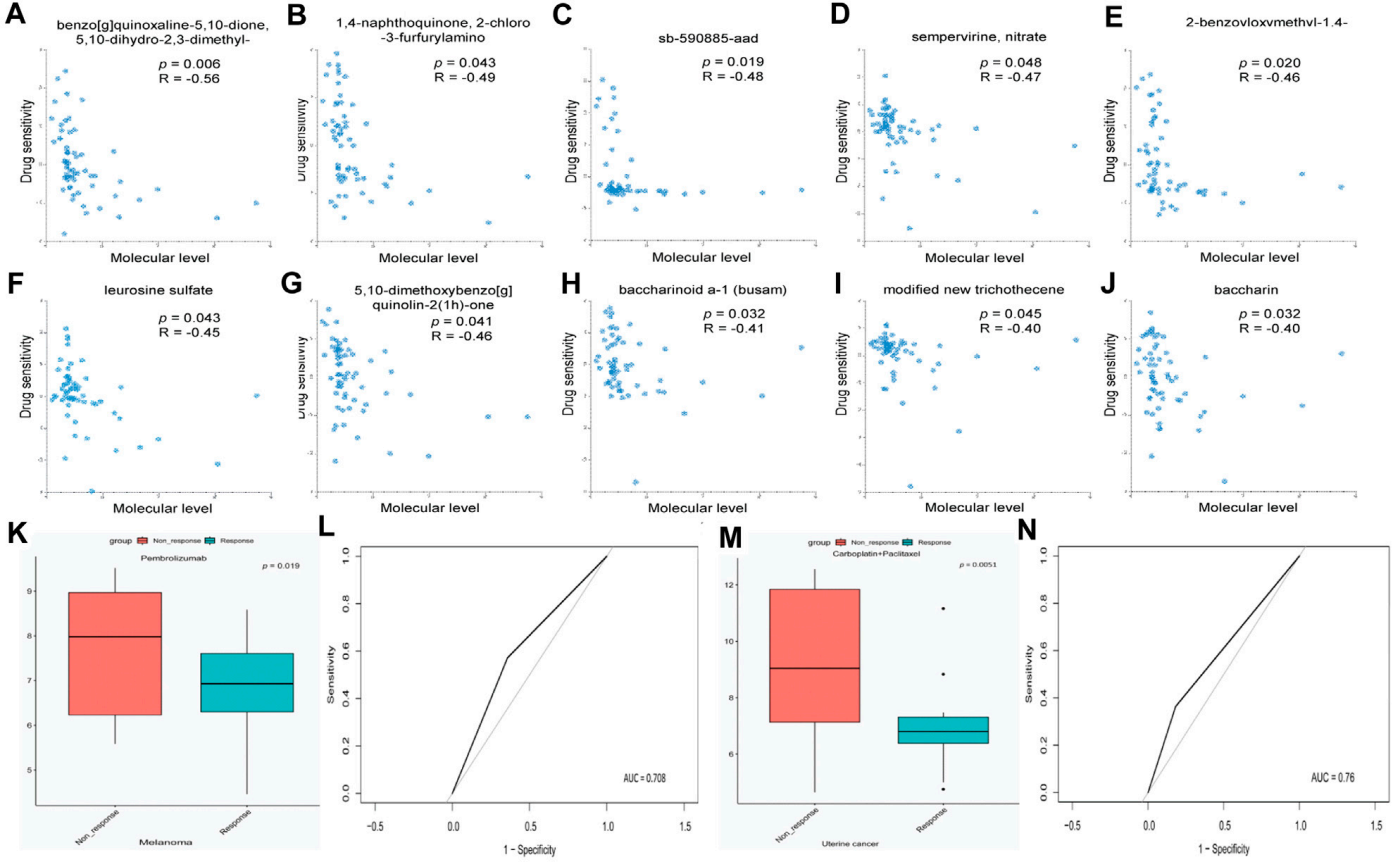
RAB42 functions as a potential drug-resistant target for certain chemotherapies. **(A–J)** Correlation analysis between drug sensitivity and mRNA expression level of Rab42 based on RNAactDrug database. **(K)** RAB42 expression in melanoma tissues with different Pembrolizumab response status. **(L)** ROC curve of RAB42 in predicting the response status of Pembrolizumab. **(M)** RAB42 expression in uterine cancer tissues with different Carboplatin + Paclitaxel response status. **(N)** ROC curve of RAB42 in predicting the response status of Carboplatin + Paclitaxel.

Considering the critical role of RAB42 in the development of pan-cancer, the role of RAB42 in drug resistance was explored. We used the RNAactDrug database to analyze the correlation between drug sensitivity and mRNA expression level of RAB42. The results showed that the drug sensitivity of benzo[g]quinoxaline-5,10-dione, 5,10-dihydro-2,3-dimethyl-, 1,4-naphthoquinone, 2-chloro -3-furfurylamino, sb-590885-aad, sempervirine, nitrate, 2-benzoyloxymethyl-1,4-naphthoquinone, leurosine sulfate, 5,10-dimethoxybenzo[g] quinolin-2(1h)-one, baccharinoid a-1 (busam), modified new trichothecene, and baccharin decreased with elevated mRNA expression level of RAB42 (Figures 6A–J). Furthermore, high expression of RAB42 was observed in the treatment group with Pembrolizumab-non-response (Figure 6K, *p* = 0.019) and in the treatment group with Carboplatin + Paclitaxel-non-response (Figure 6M, *p* = 0.0051). Moreover, RAB42 was a reliable predictor for the response to Pembrolizumab treatment (Figure 6L) and Carboplatin + Paclitaxel treatment (Figure 6N) with the AUC of 0.708 and 0.76, respectively. These results suggest that RAB42 functions as a potential drug-resistant marker to certain chemotherapies.

### Overexpression of RAB42 acts as a potential biomarker for LIHC

**FIGURE 7:**
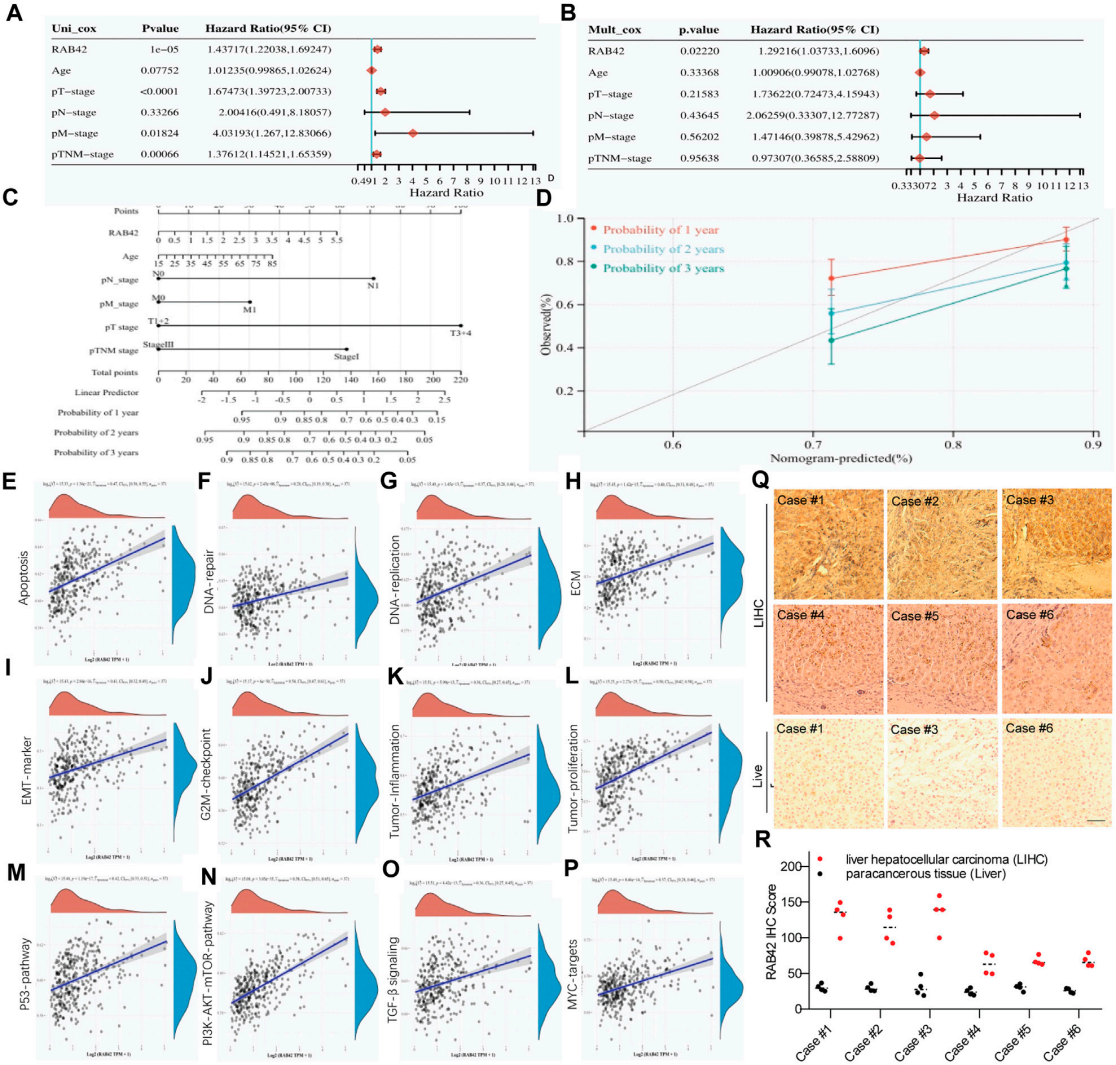
Clinical relevance of RAB42 overexpression in LIHC. **(A,B)** Univariate **(A)** and multivariate **(B)** Cox analyses the prognostic significance of RAB42 overexpression in LIHC. **(C)** Nomogram based on RAB42 expression and clinical stages. **(D)** Correction analysis diagram of the nomogram. **(E–P)** GSVA-based correlation analysis of DEGs with biological pathways. **(Q)** Representative IHC staining of RAB42 in liver cancer resection tissue. **(R)** IHC quantification of RAB42 expression in liver and LIHC samples.

Next, we explored the prognostic value of RAB42 in LIHC. Univariate and multivariate Cox regression analyses showed that RAB42 is an independent prognostic biomarker for LIHC (Figures 7A, B). To better assess patient’s prognosis in clinical practice, a nomogram was constructed as a risk model based on RAB42 expression and pathological stage (Figure 7C). Calibration curves were subsequently used to evaluate the predictive accuracy of this model for determining the prognosis of LIHC patients at 1, 2 and 3 years. The results show a favorable assessment performance (Figure 7D). To better understand the potential prognostic value of RAB42, the correlation between RAB42 expression and differentially expressed genes (DEGs) scores was calculated. The gene set variation analysis (GSVA) results showed that RAB42 overexpression is significantly correlated with that of principal genes associated with cellular apoptosis, DNA repair, DNA replication, extracellular matrix proteins (ECM), epithelial-mesenchymal transition (EMT), G2/M checkpoint, tumor-inflammation, and tumor-proliferation in LIHC (Figures 7E–L). The GSVA results also showed that RAB42 overexpression promotes the malignant phenotypes of LIHC cells mainly through the P53, PI3K-AKT-mTOR, TGF-β and MYC signaling pathways (Figures 7M–P). Moreover, high expression of RAB42 was frequently observed in surgical resected LIHC tissues (Figures 7Q, R). These results suggest that overexpressed RAB42 could be a great prognostic biomarker for LIHC.

### RAB42 overexpression or activation promotes the proliferation, migration and invasion of LIHC cells

**FIGURE 8:**
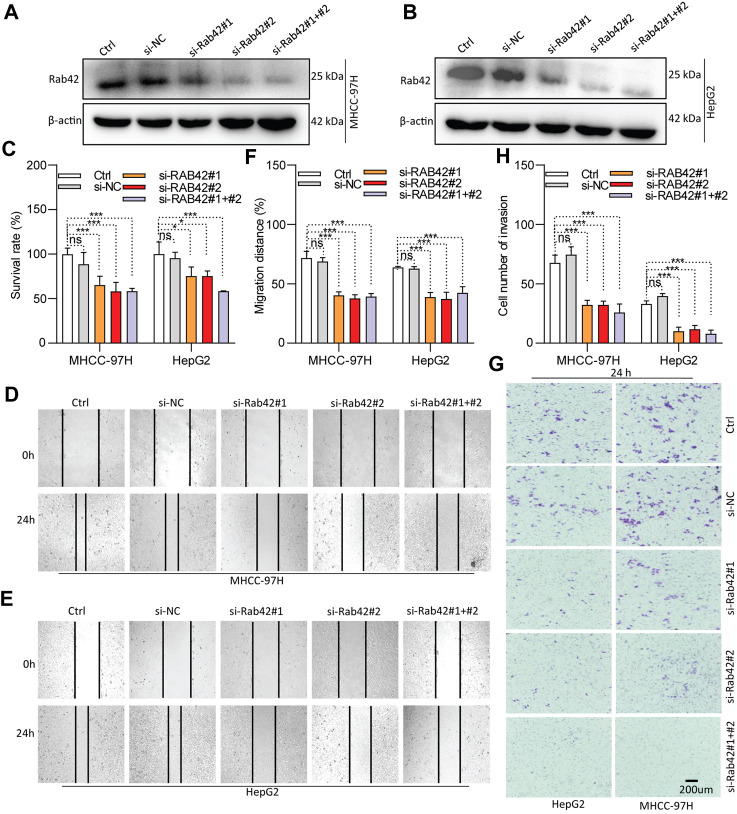
Role of RAB42 knock-down in decreasing the cellular malignancy of LIHC. **(A,B)** Determining the knockdown efficiency of RAB42 in MHCC-97H and HepG2 cells by Western blotting. **(C)** The effect of RAB42 knockdown on cellular survival determined by CCK-8 assays. **(D–F)** The effect of RAB42 knockdown on cellular migratory potential determined by wound healing assays. Representative images are shown **(D,E)**. **(G,H)** The effect of RAB42 knockdown on the cellular invasion potential determined by transwell assays. Representative images are shown **(G)**. ns, not significant; *, *p* < 0.05; ***, *p* < 0.001; *n* = 3.

**FIGURE 9:**
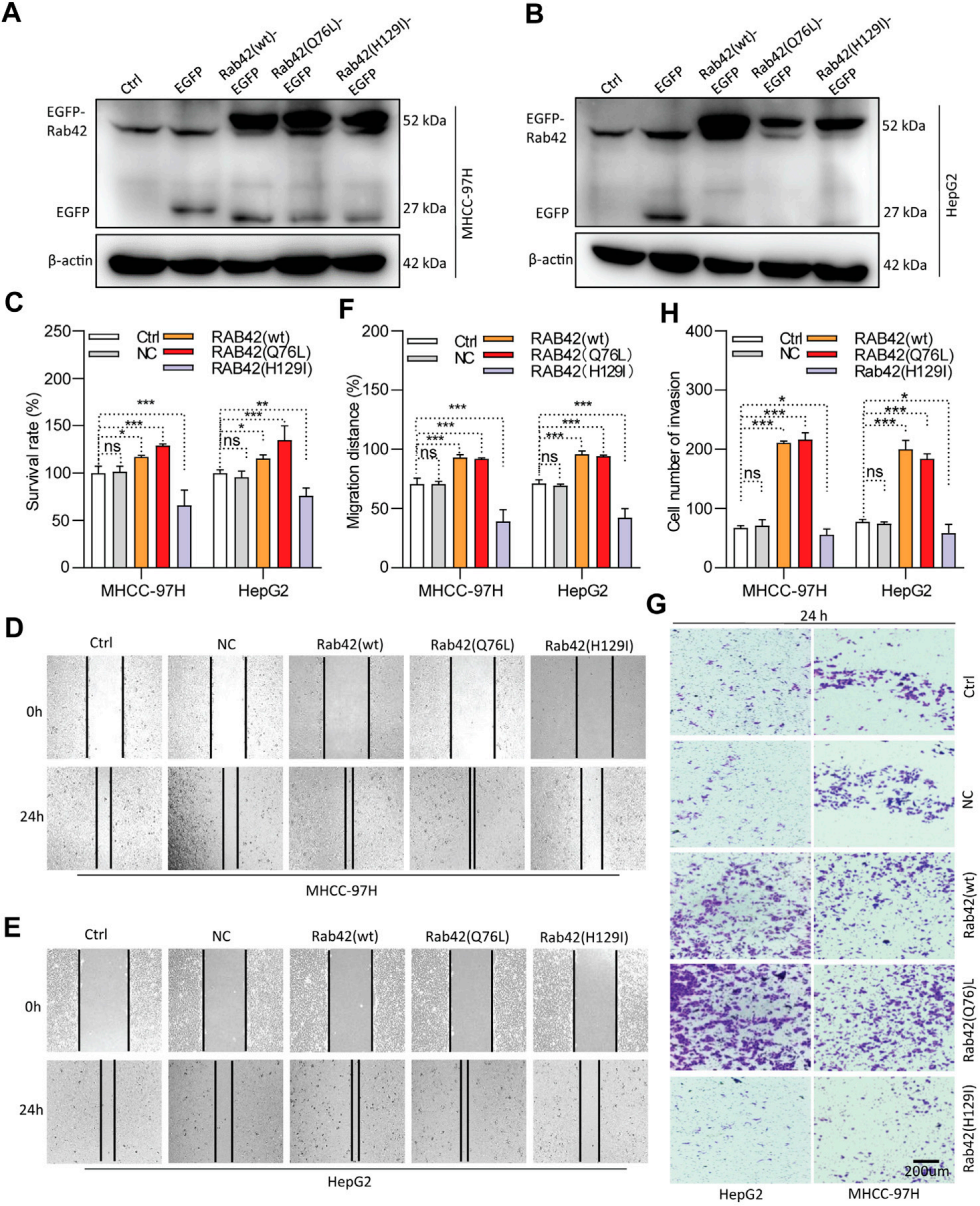
Role of RAB42 overexpression and activation in increasing the cellular malignancy of LIHC. **(A,B)** Determining the overexpression efficiency of RAB42(wt), RAB42(Q76L) and RAB42(H129I) in MHCC-97H and HepG2 cells by Western blotting. **(C)** The effect of RAB42 overexpression or activation on cellular survival determined by CCK-8 assays. **(D–F)** The effect of RAB42 overexpression or activation on cellular migratory potential determined by wound healing assays. Representative images are shown **(D,E)**. **(G,H)** The effect of RAB42 overexpression or activation on the cellular invasion potential determined by transwell assays. Representative images are shown **(G)**. ns, not significant; *p < 0.05; **p < 0.01; ***p < 0.001; *n* = 3.

As RAB42 is highly expressed and significantly associates with the prognosis of LIHC, *in vitro* experiments were conducted to investigate its biological function. Western blotting results showed that RAB42 is markedly knocked down in siRNA-transfected LIHC cells (Figures 8A,B), and significantly overexpressed in pDNA-transfected LIHC cells (Figures 9A,B). CCK-8 assays showed that the proliferation of LIHC cells is significantly decreased after RAB42 knockdown (Figure 8C). In contrast, overexpression of wild type RAB42(wt) or activated mutant RAB42(Q76L) significantly increases the cellular proliferation. Whereas overexpression of dominant-negative mutant RAB42(H129I) decreases the cell proliferation (Figure 9C). Wound healing assays showed that the migration potential of LIHC cells is significantly reduced after RAB42 knockdown (Figures 8D–F). In contrast, RAB42(wt) or RAB42(Q76L) overexpression significantly increases the cellular migration. Whereas RAB42(H129I) overexpression reduces the cellular migration (Figures 9D–F). Matrigel-based invasion assays further showed that knockdown of RAB42 expression significantly reduces the invasion potential of LIHC cells (Figures 8G, H). In contrast, RAB42(wt) or RAB42(Q76L) overexpression significantly increases the cellular invasion, while RAB42(H129I) overexpression reduces such an invasion (Figures 9G, H). Together, these results suggest that RAB42 overexpression or activation promotes the proliferation, migration and invasion of LIHC cells.

## Discussion

As key regulators of vesicle transport, RAB GTPases function as molecular switches in cell membrane trafficking process. Most RABs are expressed ubiquitously, while some of them have tissue/cell-type specificity. Different RABs localize to distinct subcellular regions or organelles, which determine the specific vesicle transport route and drive the directional membrane trafficking (Borchers et al., 2021; Homma et al., 2021; Jin et al., 2021; Wilmes and Kümmel, 2023). Emerging evidence showed that aberrant expression of RAB GTPases is closely associated with tumorigenesis (Tzeng and Wang, 2016; Yang C. C. et al., 2021; Jin et al., 2021). Partial RAB GTPases promote tumorigenesis and tumor progression by cooperating with oncogenic signaling. Moreover, uncontrolled interaction between RAB GTPases and their regulators, such as guanine nucleotide exchange factors (GEFs), GTPase activating proteins (GAPs), GDP-dissociation inhibitors (GDIs), GDI displacement factors (GDFs) and tethering factors, could modulate the malignancies. However, a minor fraction of RAB GTPases are proposed to serve as tumor suppressors, and posttranslational modifications may affect their GTPase activity (Tzeng and Wang, 2016; Jin et al., 2021). Thus, RAB proteins exert distinct functions depending on the types or subtypes of cancers.

Since RAB42 has recently been identified as RAB7b (Marubashi and Fukuda, 2020), its biological function is supposed to control the vesicular trafficking from endosomes to trans-Golgi network (TGN). RAB42 acts as a negative regulator of TLR9 and TLR4 signaling. It not only suppresses TLR9-triggered TNFA, IL6, and IFNB production, but also promotes the lysosomal degradation of TLR4 in macrophages (Progida et al., 2010). RAB42 also promotes megakaryocytic differentiation by increasing the NF-kappaB-dependent IL6 production and enhancing the STAT3 associated with GATA1 (He et al., 2011). A few studies demonstrated that RAB42 is expressed in mucinous lung adenocarcinoma, breast cancer, glioma, and LIHC (Di Carlo et al., 2020; Yang Y. et al., 2021; Liu et al., 2021; Kong et al., 2023). Here, our results revealed that RAB42 overexpression occurs in pan-cancer (Figures 1A, B), particularly in advanced pathological stages of most cancer types (Figure 1C). The first major finding of our study is that RAB42 overexpression is markedly correlated with prognostic indicators (OS, PFS, DFS and DSS) in most cancers (Figures 2A–D; Supplementary Figures S1–S4). These results are partially consistent with previous studies showing that RAB42 could serve as a prognostic marker for glioblastoma (GBM) (Sun et al., 2022), liver cancer (Peng et al., 2022) and some other types of cancers (Zheng et al., 2022). These findings support that overexpression of RAB42 could serve as a diagnostic and prognostic biomarker for pan-cancer.

The second major finding in our study is that RAB42 overexpression significantly affects the immune cell function and infiltration patterns across various cancer types (Figures 3, 4). Overexpression of RAB42 is correlated with the contents of immune infiltration, types of immune-infiltrating cells, and expression of immune checkpoints. These results mirror well with the results from other studies (Yang C. C. et al., 2021; Peng et al., 2022; Kong et al., 2023). Previous study shown that RAB42 is positively correlated with exhausted CD8^+^ T cells in liver cancer (Peng et al., 2022). These results suggest that although RAB42 promotes the infiltration of immune cells, it could induce the formation of immunosuppressive milieu by stimulating the expression of immune checkpoints in pan-cancer.

Tumor progression and treatment resistance are affected by immune-infiltrating cells. For example, Treg cells are associated with poor prognosis in cancers (Tanaka and Sakaguchi, 2019). The significant correlation between RAB42 overexpression and Treg cell level implies that RAB42 plays an essential role in tumor microenvironment. ICIs have been used for clinical therapy but have unsatisfied efficacy. TMB, HRD and LOH are frequently used to predict the therapeutic efficacy of ICIs (Sharma et al., 2021). The significant correlation of RAB42 overexpression with these predictive markers of ICIs in SARC, ACC, LUAD and KICH strongly support the potential of RAB42 as a new predictive marker for ICIs treatment. Additionally, the correlation network analysis also indicates that RAB42 is a critical regulator that promotes the expression of m6A RNA MRGs and stemness genes (Figure 5). These results are essential because previous studies suggested that RAB42 is involved in immune escape by modulating PD-L1 expression (Kong et al., 2023). These findings support the prognostic value of RAB42 in TIME.

RAB GTPases could also serve as biomarkers for resistance to certain small molecule chemotherapeutic drugs (Wei et al., 2022; Li C. et al., 2023). For example, RAB1b is reported to be involved in sorafenib-resistance of HCC (Xu et al., 2023), and high expression of RAB26 promotes the cisplatin-based drug resistance in lung cancer (Wang et al., 2023). Here, we analyze the role of RAB42 in drug resistance in pan-cancer. We found that high expression level of RAB42 induces chemotherapeutic drug-resistance in pan-cancer (Figure 6). Paclitaxel plus carboplatin is the standard first-line chemotherapy for endometrial cancer (Eskander et al., 2023). Interestingly, patients with high expression of RAB42 seem to be resistant to the treatment of paclitaxel plus carboplatin. This phenomena suggests that targeting RAB42 might well improve the therapeutic efficiency of carboplatin plus paclitaxel in uterine cancer. Whether RAB42 is a resistant target of paclitaxel plus carboplatin in pan-cancer (especially in LIHC) warrants further investigation in the future.

Accumulating studies suggest that RAB GTPases can serve as promising therapeutic targets for HCC treatment (Yang C. C. et al., 2021). Here, our study reveals the prognostic value of RAB42 in LIHC and highlights that RAB42 could serve as a driver for LIHC development (Figure 7). Knockdown of RAB42 expression or deactivation of its function markedly inhibits the proliferation, invasion and migration of LIHC cells (Figures 8, 9). Overexpression of RAB42 or activation of its function significantly promotes these cellular malignant phenotypes (Figures 8, 9). These results support the recent findings that RAB42 may act as a pro-oncoprotein that promotes tumor progression, reshapes tumor microenvironment, and could be a new diagnostic and therapeutic marker for LIHC (Paul and Mukherjee, 1986; Kong et al., 2023). Specifically, through regulating the vesicle trafficking process, RAB42 overexpression play crucial roles in HCC pathological processes, including cell survival, proliferation, motility, metastasis and invasion. However, it is worth noted that there is functional redundancy of the RAB GTPase family.

## Conclusion

In summary, our study highlights the prognostic, and chemoresistance-predictive values of RAB42. RAB42 overexpression correlated with RNA modification, immune cell infiltration, immunotherapeutic and chemotherapeutic responses. RAB42 could serve as a prognostic marker and therapeutic target in pan-cancer, especially in LIHC.

## Data Availability

The original contributions presented in the study are included in the article/Supplementary Material, further inquiries can be directed to the corresponding author.
