# Supplementary material for: RAB42 overexpression correlates with poor prognosis, immune cell infiltration and chemoresistance
Source: Front Pharmacol. 2024 Jul 19;15:1445170. doi: 10.3389/fphar.2024.1445170 (PMC11294155; doi:10.3389/fphar.2024.1445170)
Supplement: Supplementary file 1 [file DataSheet1.docx]

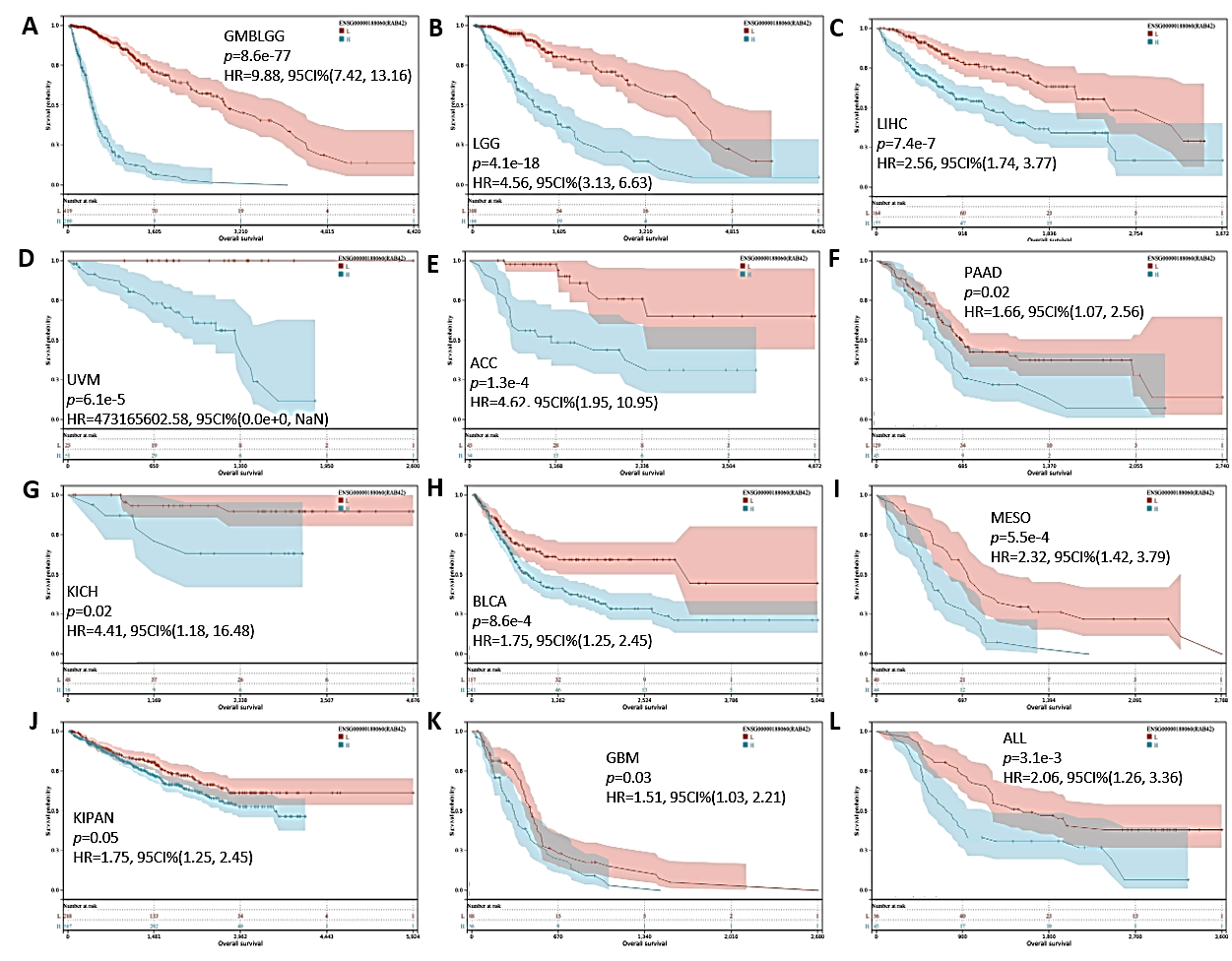
**Figure S1** Kaplan–Meier curves revealing the correlation between Rab42 overexpression and OS. **A** GBMLGG, **B** LGG, **C** LIHC, **D** UVM, **E** ACC, **F** PAAD, **G** KICH, **H** BLCA, **I** MESO, **J** KIPAN, **K** GBM, **L** ALL. OS, overall survival.


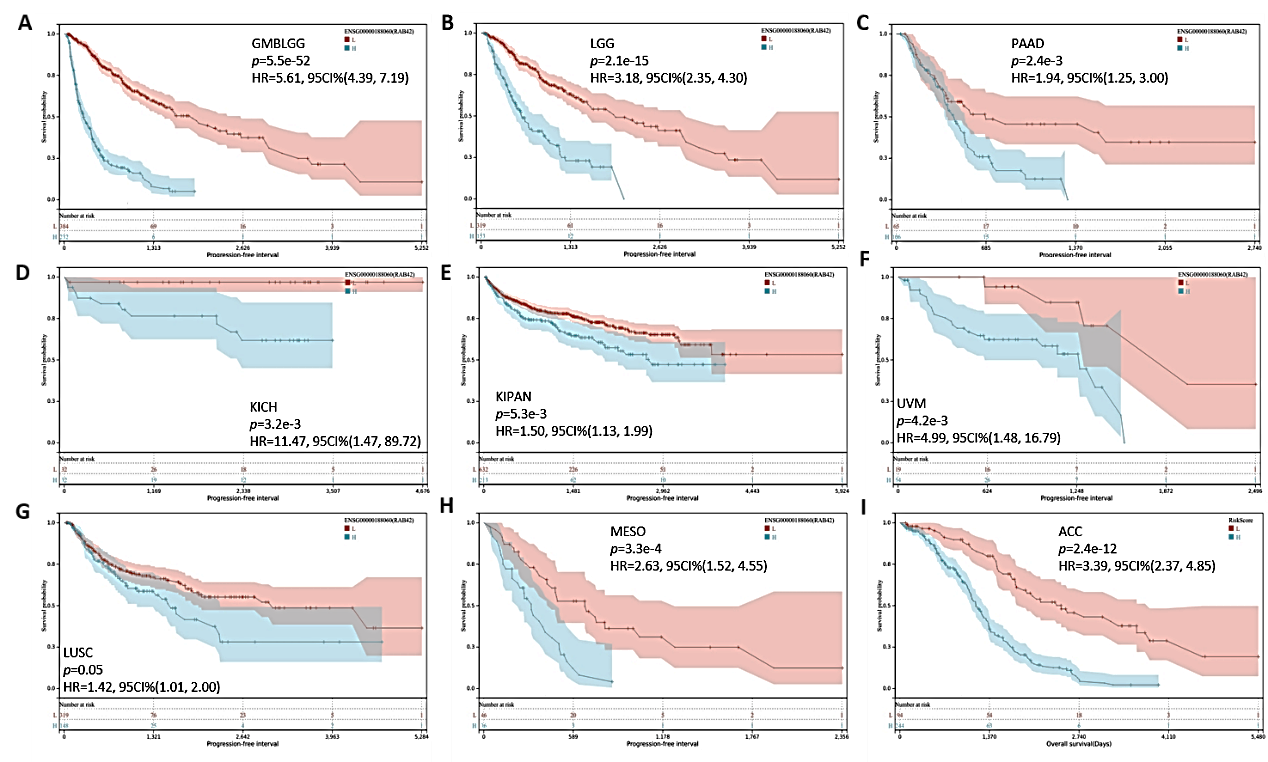
 **Figure S2** Kaplan–Meier curves revealing the correlation between Rab42 overexpression and PFS.  **A** GBMLGG, **B** LGG, **C** PAAD, **D** KICH, **E** KIPAN, **F** UVM, **G** LUSC, **H** MESO, **I** ACC. PFS, progression-free survival.


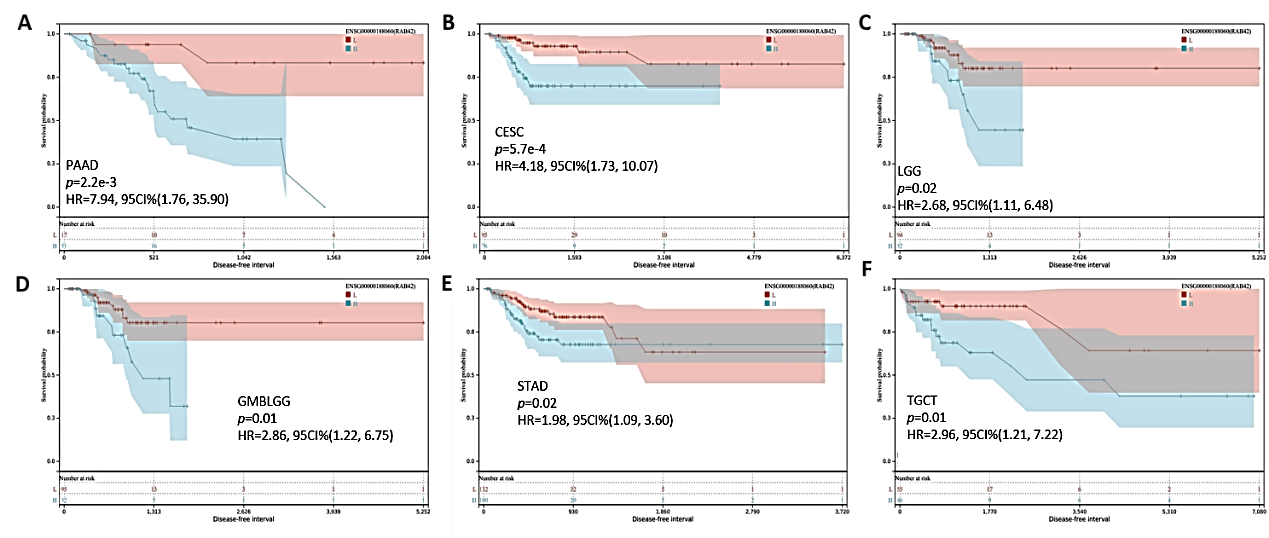
 **Figure S3** Kaplan–Meier curves revealing the correlation between Rab42 overexpression and DFS. **A** PAAD, **B** CESC, **C** LGG, **D** GBMLGG, **E** STAD, **F** TGCT. DFS, disease-free survival.


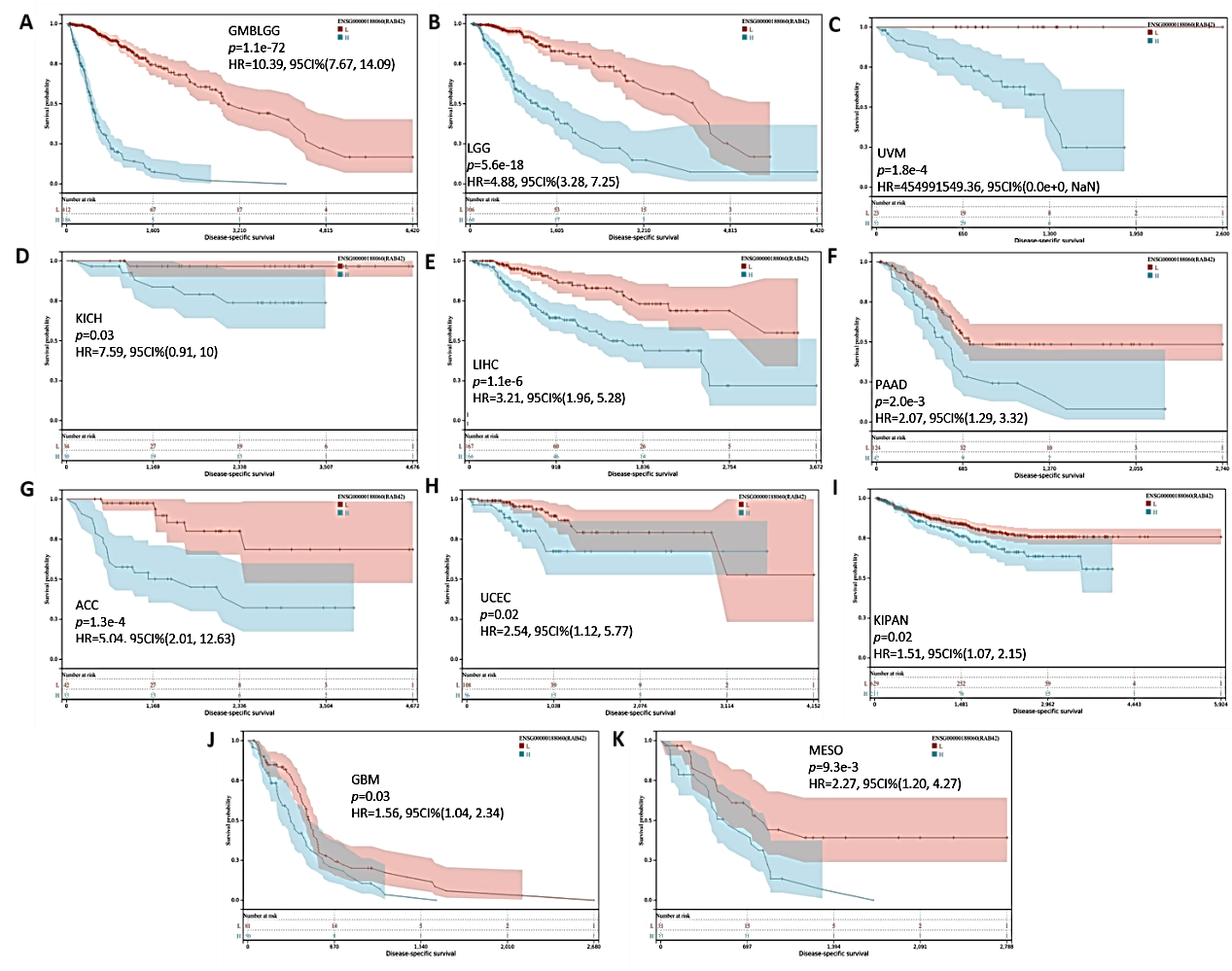
 **Figure S4** Kaplan-Meier curves revealing the correlation between Rab42 overexpression and DSS. **A** GBMLGG, **B** LGG, **C** UVM, **D** KICH, **E** LIHC, **F** PAAD, **G** ACC, **H** UCEC, **I** KIPAN, **J** GBM, **K** MESO. DSS, disease-specific survival.


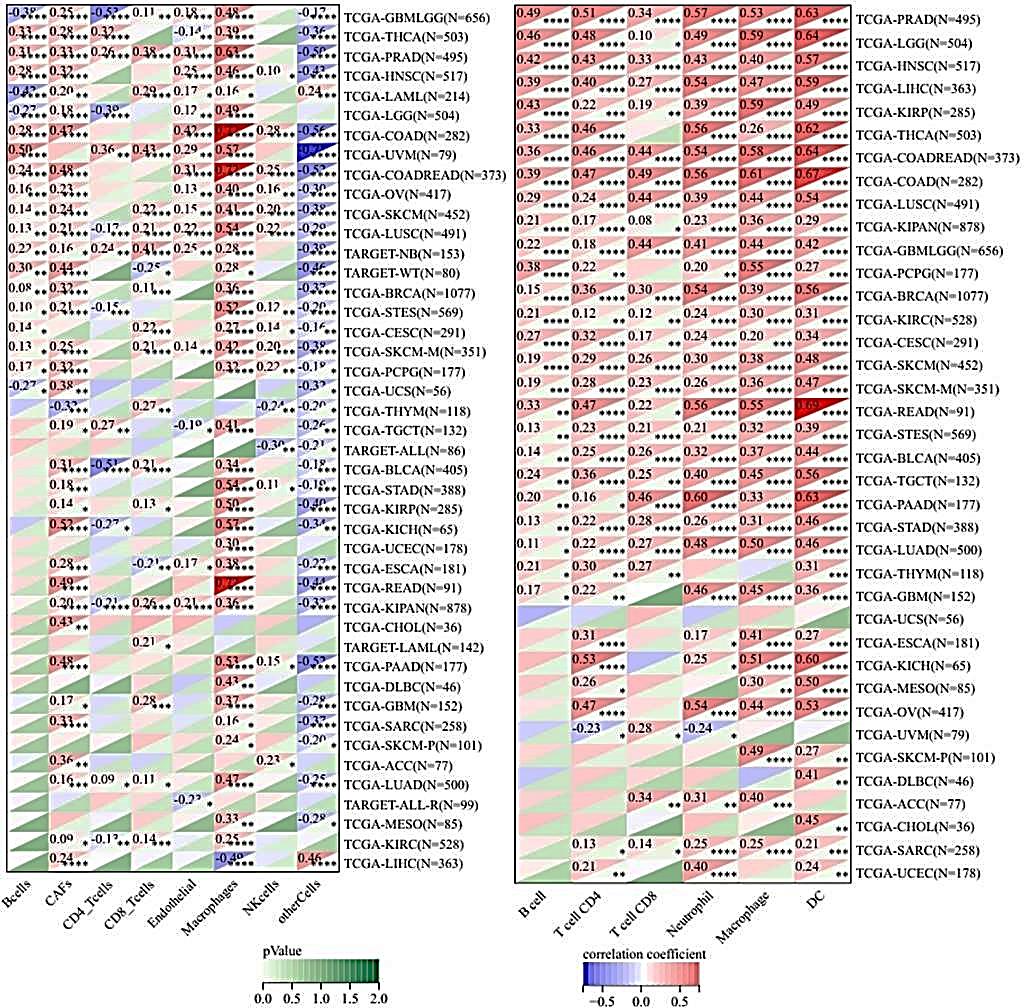


**Figure S5** Correlation of Rab42 overexpression with the infiltration content of immune cells. Rab42 expression level was closely correlated with immune cell infiltration in cancers, as determined via **A** EPIC and **B** TIMER algorithms. * *p*<0.05; ** *p*<0.01; *** *p*<0.001; **** *p*<0.0001.

**Table S1**  The abbreviations in this study

| **Abbreviations** | **Full name** |
| --- | --- |
| ACC | Adrenocortical carcinoma |
| BLCA | Bladder urothelial carcinoma |
| BRCA | Breast invasive carcinoma |
| CESC | Cervical squamous cell carcinoma and endocervical adenocarcinoma |
| CHOL | Cholangiocarcinoma |
| COAD | Colon adenocarcinoma |
| COADREAD | Colon adenocarcinoma/Rectum adenocarcinoma Esophageal carcinoma |
| DLBC | Diffuse large B-cell lymphoma |
| ESCA | Esophageal carcinoma |
| GBM | Glioblastoma multiforme |
| GBMLGG | Glioblastoma and low-grade glioma |
| HNSC | Head and neck squamous cell carcinoma |
| KICH | Kidney chromophobe carcinoma |
| KIPAN | Pan-kidney cohort (KICH+KIRC+KIRP) |
| KIRC | Kidney renal clear cell carcinoma |
| KIRP | Kidney renal papillary cell carcinoma |
| LAML | Acute myeloid leukemia-like |
| LGG | Lower-grade glioma |
| LIHC | Liver hepatocellular carcinoma |
| LUAD | Lung adenocarcinoma |
| LUSC | Lung squamous cell carcinoma |
| MESO | Mesothelioma |
| SOC | Serous ovarian cancer |
| PAAD | Pancreatic adenocarcinoma |
| PCPG | Pheochromocytoma and paraganglioma |
| PRAD | Prostate adenocarcinoma |
| READ | Rectum adenocarcinoma |
| SARC | Sarcoma |
| SKCM | Skin cutaneous melanoma |
| STAD | Stomach adenocarcinoma |
| STES | Stomach and esophageal carcinoma |
| TGCT | Testicular germ cell tumors |
| THCA | Thyroid carcinoma |
| THYM | Thymoma |
| UCEC | Uterine corpus endometrial carcinoma |
| UCS | Uterine carcinosarcoma |
| UVM | Uveal melanoma |
| RAB42 | Ras-associated binding protein 42 |
| TCGA | The cancer genome atlas |
| GTEx | Genotype-tissue expression |
| CCLE | Cancer cell line encyclopedia |
